# Supplementary material for: Pyruvate Dehydrogenase Kinase 1 inhibition mediated oxidative phosphorylation enhancement in cartilage promotes osteoarthritis progression
Source: BMC Musculoskelet Disord. 2023 Jul 20;24:597. doi: 10.1186/s12891-023-06585-6 (PMC10357736; doi:10.1186/s12891-023-06585-6)
Supplement: Supplementary file 1 — Additional file 1. [file 12891_2023_6585_MOESM1_ESM.zip › KEGG.Gsea.1653622432464/KEGG_PATHOGENIC_ESCHERICHIA_COLI_INFECTION.html]

Details for gene set KEGG\_PATHOGENIC\_ESCHERICHIA\_COLI\_INFECTION[GSEA]

|  || Dataset | 55457\_collapsed\_to\_symbols.cls.cls #OA\_versus\_normal.cls.cls #OA\_versus\_normal\_repos |
| Phenotype | cls.cls#OA\_versus\_normal\_repos |
| Upregulated in class | normal |
| GeneSet | KEGG\_PATHOGENIC\_ESCHERICHIA\_COLI\_INFECTION |
| Enrichment Score (ES) | -0.37447327 |
| Normalized Enrichment Score (NES) | -1.44059 |
| Nominal p-value | 0.046511628 |
| FDR q-value | 0.21790273 |
| FWER p-Value | 0.979 |
Table: GSEA Results Summary

  

Fig 1: Enrichment plot: KEGG\_PATHOGENIC\_ESCHERICHIA\_COLI\_INFECTION      
 Profile of the Running ES Score & Positions of GeneSet Members on the Rank Ordered List

  

| SYMBOL | TITLE | RANK IN GENE LIST | RANK METRIC SCORE | RUNNING ES | CORE ENRICHMENT || 1 | ARPC1A | actin related protein 2/3 complex subunit 1A [Source:HGNC Symbol;Acc:HGNC:703] | 47 | 1.003 | 0.0490 | No |
| 2 | NCK1 | NCK adaptor protein 1 [Source:HGNC Symbol;Acc:HGNC:7664] | 222 | 0.804 | 0.0776 | No |
| 3 | ARPC5 | actin related protein 2/3 complex subunit 5 [Source:HGNC Symbol;Acc:HGNC:708] | 1118 | 0.492 | 0.0338 | No |
| 4 | ARPC3 | actin related protein 2/3 complex subunit 3 [Source:HGNC Symbol;Acc:HGNC:706] | 1743 | 0.393 | 0.0059 | No |
| 5 | CD14 | CD14 molecule [Source:HGNC Symbol;Acc:HGNC:1628] | 1928 | 0.369 | 0.0109 | No |
| 6 | TUBA3E | tubulin alpha 3e [Source:HGNC Symbol;Acc:HGNC:20765] | 2169 | 0.342 | 0.0102 | No |
| 7 | ARPC1B | actin related protein 2/3 complex subunit 1B [Source:HGNC Symbol;Acc:HGNC:704] | 2184 | 0.340 | 0.0270 | No |
| 8 | CDH1 | cadherin 1 [Source:HGNC Symbol;Acc:HGNC:1748] | 2589 | 0.292 | 0.0108 | No |
| 9 | TUBB7P | tubulin beta 7 pseudogene [Source:HGNC Symbol;Acc:HGNC:12413] | 2744 | 0.275 | 0.0133 | No |
| 10 | ARPC2 | actin related protein 2/3 complex subunit 2 [Source:HGNC Symbol;Acc:HGNC:705] | 2893 | 0.261 | 0.0155 | No |
| 11 | CLDN1 | claudin 1 [Source:HGNC Symbol;Acc:HGNC:2032] | 3089 | 0.241 | 0.0130 | No |
| 12 | TLR5 | toll like receptor 5 [Source:HGNC Symbol;Acc:HGNC:11851] | 3261 | 0.225 | 0.0115 | No |
| 13 | TUBB | tubulin beta class I [Source:HGNC Symbol;Acc:HGNC:20778] | 3296 | 0.221 | 0.0204 | No |
| 14 | LY96 | lymphocyte antigen 96 [Source:HGNC Symbol;Acc:HGNC:17156] | 3630 | 0.191 | 0.0045 | No |
| 15 | PRKCA | protein kinase C alpha [Source:HGNC Symbol;Acc:HGNC:9393] | 3728 | 0.183 | 0.0066 | No |
| 16 | EZR | ezrin [Source:HGNC Symbol;Acc:HGNC:12691] | 3817 | 0.174 | 0.0089 | No |
| 17 | TUBB2B | tubulin beta 2B class IIb [Source:HGNC Symbol;Acc:HGNC:30829] | 4777 | 0.089 | -0.0611 | No |
| 18 | TUBAL3 | tubulin alpha like 3 [Source:HGNC Symbol;Acc:HGNC:23534] | 5043 | 0.070 | -0.0781 | No |
| 19 | TUBA8 | tubulin alpha 8 [Source:HGNC Symbol;Acc:HGNC:12410] | 5208 | 0.056 | -0.0879 | No |
| 20 | TUBB1 | tubulin beta 1 class VI [Source:HGNC Symbol;Acc:HGNC:16257] | 5561 | 0.028 | -0.1138 | No |
| 21 | YWHAZ | tyrosine 3-monooxygenase/tryptophan 5-monooxygenase activation protein zeta [Source:HGNC Symbol;Acc:HGNC:12855] | 5770 | 0.010 | -0.1295 | No |
| 22 | TUBB4A | tubulin beta 4A class IVa [Source:HGNC Symbol;Acc:HGNC:20774] | 5801 | 0.008 | -0.1314 | No |
| 23 | WAS | WASP actin nucleation promoting factor [Source:HGNC Symbol;Acc:HGNC:12731] | 6666 | -0.054 | -0.1958 | No |
| 24 | ARPC5L | actin related protein 2/3 complex subunit 5 like [Source:HGNC Symbol;Acc:HGNC:23366] | 7023 | -0.082 | -0.2192 | No |
| 25 | KRT18 | keratin 18 [Source:HGNC Symbol;Acc:HGNC:6430] | 7027 | -0.082 | -0.2152 | No |
| 26 | TUBB4B | tubulin beta 4B class IVb [Source:HGNC Symbol;Acc:HGNC:20771] | 7370 | -0.108 | -0.2361 | No |
| 27 | HCLS1 | hematopoietic cell-specific Lyn substrate 1 [Source:HGNC Symbol;Acc:HGNC:4844] | 7653 | -0.131 | -0.2512 | No |
| 28 | TUBA4A | tubulin alpha 4a [Source:HGNC Symbol;Acc:HGNC:12407] | 7668 | -0.132 | -0.2453 | No |
| 29 | TLR4 | toll like receptor 4 [Source:HGNC Symbol;Acc:HGNC:11850] | 7703 | -0.134 | -0.2409 | No |
| 30 | RHOA | ras homolog family member A [Source:HGNC Symbol;Acc:HGNC:667] | 8601 | -0.201 | -0.3002 | No |
| 31 | YWHAQ | tyrosine 3-monooxygenase/tryptophan 5-monooxygenase activation protein theta [Source:HGNC Symbol;Acc:HGNC:12854] | 8675 | -0.207 | -0.2950 | No |
| 32 | ABL1 | "ABL proto-oncogene 1, non-receptor tyrosine kinase [Source:HGNC Symbol;Acc:HGNC:76]" | 9081 | -0.238 | -0.3140 | No |
| 33 | ARPC4 | actin related protein 2/3 complex subunit 4 [Source:HGNC Symbol;Acc:HGNC:707] | 9522 | -0.276 | -0.3338 | No |
| 34 | NCK2 | NCK adaptor protein 2 [Source:HGNC Symbol;Acc:HGNC:7665] | 9755 | -0.297 | -0.3362 | No |
| 35 | FYN | "FYN proto-oncogene, Src family tyrosine kinase [Source:HGNC Symbol;Acc:HGNC:4037]" | 10092 | -0.330 | -0.3451 | No |
| 36 | TUBB6 | tubulin beta 6 class V [Source:HGNC Symbol;Acc:HGNC:20776] | 10471 | -0.370 | -0.3550 | Yes |
| 37 | ROCK2 | Rho associated coiled-coil containing protein kinase 2 [Source:HGNC Symbol;Acc:HGNC:10252] | 10648 | -0.388 | -0.3484 | Yes |
| 38 | ARHGEF2 | Rho/Rac guanine nucleotide exchange factor 2 [Source:HGNC Symbol;Acc:HGNC:682] | 10808 | -0.406 | -0.3394 | Yes |
| 39 | TUBA3C | tubulin alpha 3c [Source:HGNC Symbol;Acc:HGNC:12408] | 10884 | -0.415 | -0.3235 | Yes |
| 40 | TUBB2A | tubulin beta 2A class IIa [Source:HGNC Symbol;Acc:HGNC:12412] | 10972 | -0.427 | -0.3079 | Yes |
| 41 | CTTN | cortactin [Source:HGNC Symbol;Acc:HGNC:3338] | 11677 | -0.528 | -0.3349 | Yes |
| 42 | TUBA1B | tubulin alpha 1b [Source:HGNC Symbol;Acc:HGNC:18809] | 11888 | -0.569 | -0.3214 | Yes |
| 43 | TUBA1A | tubulin alpha 1a [Source:HGNC Symbol;Acc:HGNC:20766] | 11974 | -0.589 | -0.2971 | Yes |
| 44 | ROCK1 | Rho associated coiled-coil containing protein kinase 1 [Source:HGNC Symbol;Acc:HGNC:10251] | 12046 | -0.605 | -0.2709 | Yes |
| 45 | TUBA1C | tubulin alpha 1c [Source:HGNC Symbol;Acc:HGNC:20768] | 12129 | -0.626 | -0.2444 | Yes |
| 46 | WASL | WASP like actin nucleation promoting factor [Source:HGNC Symbol;Acc:HGNC:12735] | 12207 | -0.647 | -0.2164 | Yes |
| 47 | NCL | nucleolin [Source:HGNC Symbol;Acc:HGNC:7667] | 12255 | -0.662 | -0.1853 | Yes |
| 48 | ITGB1 | integrin subunit beta 1 [Source:HGNC Symbol;Acc:HGNC:6153] | 12566 | -0.780 | -0.1685 | Yes |
| 49 | ACTG1 | actin gamma 1 [Source:HGNC Symbol;Acc:HGNC:144] | 12613 | -0.806 | -0.1297 | Yes |
| 50 | CDC42 | cell division cycle 42 [Source:HGNC Symbol;Acc:HGNC:1736] | 12681 | -0.852 | -0.0902 | Yes |
| 51 | ACTB | actin beta [Source:HGNC Symbol;Acc:HGNC:132] | 12757 | -0.920 | -0.0477 | Yes |
| 52 | CTNNB1 | catenin beta 1 [Source:HGNC Symbol;Acc:HGNC:2514] | 12866 | -1.119 | 0.0026 | Yes |
Table: GSEA details [plain text format]

  

Fig 2: KEGG\_PATHOGENIC\_ESCHERICHIA\_COLI\_INFECTION      
 Blue-Pink O' Gram in the Space of the Analyzed GeneSet

  

Fig 3: KEGG\_PATHOGENIC\_ESCHERICHIA\_COLI\_INFECTION: Random ES distribution      
 Gene set null distribution of ES for **KEGG\_PATHOGENIC\_ESCHERICHIA\_COLI\_INFECTION**
